# Implementation and Evaluation of a Digitally Enabled Precision Public Health Intervention to Reduce Inappropriate Gabapentinoid Prescription: Cluster Randomized Controlled Trial

**DOI:** 10.2196/33873

**Published:** 2022-01-10

**Authors:** Andre Q Andrade, Jean-Pierre Calabretto, Nicole L Pratt, Lisa M Kalisch-Ellett, Gizat M Kassie, Vanessa T LeBlanc, Emmae Ramsay, Elizabeth E Roughead

**Affiliations:** 1 Quality Use of Medicines and Pharmacy Research Centre UniSA Clinical and Medical Sciences University of South Australia Adelaide Australia; 2 Australian Medicines Handbook Pty Ltd Adelaide Australia

**Keywords:** audit and feedback, digital health, precision public health, digital intervention, primary care, physician, health professional, health education

## Abstract

**Background:**

Digital technologies can enable rapid targeted delivery of audit and feedback interventions at scale. Few studies have evaluated how mode of delivery affects clinical professional behavior change and none have assessed the feasibility of such an initiative at a national scale.

**Objective:**

The aim of this study was to develop and evaluate the effect of audit and feedback by digital versus postal (letter) mode of delivery on primary care physician behavior.

**Methods:**

This study was developed as part of the Veterans’ Medicines Advice and Therapeutics Education Services (MATES) program, an intervention funded by the Australian Government Department of Veterans’ Affairs that provides targeted education and patient-specific audit with feedback to Australian general practitioners, as well as educational material to veterans and other health professionals. We performed a cluster randomized controlled trial of a multifaceted intervention to reduce inappropriate gabapentinoid prescription, comparing digital and postal mode of delivery. All veteran patients targeted also received an educational intervention (postal delivery). Efficacy was measured using a linear mixed-effects model as the average number of gabapentinoid prescriptions standardized by defined daily dose (individual level), and number of veterans visiting a psychologist in the 6 and 12 months following the intervention.

**Results:**

The trial involved 2552 general practitioners in Australia and took place in March 2020. Both intervention groups had a significant reduction in total gabapentinoid prescription by the end of the study period (digital: mean reduction of 11.2%, *P*=.004; postal: mean reduction of 11.2%, *P*=.001). We found no difference between digital and postal mode of delivery in reduction of gabapentinoid prescriptions at 12 months (digital: –0.058, postal: –0.058, *P*=.98). Digital delivery increased initiations to psychologists at 12 months (digital: 3.8%, postal: 2.0%, *P*=.02).

**Conclusions:**

Our digitally delivered professional behavior change intervention was feasible, had comparable effectiveness to the postal intervention with regard to changes in medicine use, and had increased effectiveness with regard to referrals to a psychologist. Given the logistical benefits of digital delivery in nationwide programs, the results encourage exploration of this mode in future interventions.

## Introduction

Audit and feedback interventions can be effective tools to promote evidence translation through professional behavior change [1]. Audit and feedback interventions objectively measure professional performance and create benchmarks against professional standards. Development and dissemination of audit and feedback interventions have benefited from advances in information technology that have increased data availability, decreased costs, and improved automation. As a cost-effective and data-driven intervention, audit and feedback seems well suited for migrating to a fully electronic mode of delivery [2].

Despite the potential advantages of electronic delivery, there is a theoretical and evidence gap regarding the influence of changing the mode of delivery on the efficacy of behavior change interventions [3]. Previous studies of behavior change interventions suggest that the mode of delivery may influence the efficacy of behavior change techniques. The most likely mechanism is a fundamental change in user experience, which may elicit different responses [3]. Use of different modes of delivery changes users’ experiences by creating new contexts (eg, SMS text message sent at any given time versus scheduled educational sessions), creating more personal experiences (eg, face-to-face group sessions versus social media), and providing new modes of interaction (eg, interactive computer interventions versus printed material). A review on the use of behavior change techniques for smoking cessation found a positive effect of the techniques when delivered in person, but not when delivered in writing [4]. Another review on internet-delivered behavior change interventions found increased efficacy when using additional modes of delivery, such as SMS text messages and email communication [5]. However, further analysis of the same data set did not find a synergistic effect between any combination of mode of delivery and behavior change technique. The authors suggest that having additional channels of delivery may be beneficial, but were unable to recommend which modes to use for particular behavior change techniques [6].

Evidence on the influence of digital delivery in audit and feedback’s efficacy is also needed. A 2017 review of electronic audit and feedback interventions found heterogeneous results due to differences in the intervention implementation and the underlying theory and context [7].

Following the suggestions put forward by [8], the aim of this study was to evaluate the influence of delivering an audit and feedback intervention by secure digital delivery to the clinical desktop for integration to the patient care record, and compare it to the same intervention delivered by post. The behavior change goal was the reduction in gabapentinoid prescription. Gabapentinoids are a group of medicines that includes gabapentin and pregabalin. Evidence suggests these medicines are often incorrectly prescribed in nonneuropathic pain [9], with significant risk of serious side effects and potential for abuse and misuse [10]. To test the efficacy of the digital intervention, we (the authors) performed a cluster randomized trial of an intervention aimed at reducing inappropriate prescription of gabapentinoids by primary care providers.

## Methods

### The Veterans’ Medicines Advice and Therapeutics Education Services Program

The study is part of the Veterans’ Medicines Advice and Therapeutics Education Services (MATES) program [11], which is funded by the Australian Government Department of Veterans’ Affairs and provides medicines advice and promotes physician adoption of best practices. Since 2004, it has provided repeated multifaceted interventions, composed of an audit and feedback and educational component targeted at general practitioners (GPs), with supportive educational material provided to veterans, pharmacists, and other health professionals. The intervention is informed by social cognitive theory [12], the transtheoretical model [13], and the health promotion model PRECEDE-PROCEED [14]. Between 2004 and 2021, the program delivered 62 distinct interventions to GPs and veterans in all Australian states.

The intervention is developed in three sequential steps. The first is an epidemiological analysis performed on a comprehensive database containing administrative health claims data collected by the Australian Government Department of Veterans’ Affairs (DVA). The DVA claims database includes all health care services and medicines funded by DVA, including outpatient and hospital services, aged care, prescription medicines, allied health services, and other health coordination and support services.

The second step is the design of the educational component. It involves clinicians, researchers, and veterans, and results in two sets of educational materials. The first is targeted at GPs, and describes scientific updates and therapeutic recommendations. The second is targeted at veterans, and promotes general awareness and practical guidelines for patients.

The third step is the development of the audit and feedback component. The design process is also collaborative. The intervention adopts evidence-based strategies listed in [15] to improve effectiveness, such as authority (content endorsed by a clinical DVA committee), focus on problems with larger scope for improvement, and repeated feedback (topics are revisited after a few years). It also incorporates behavior change techniques such as heuristic techniques, goal setting, and prompts, which have been shown to improve perceived usefulness [16].

### Digital Solution Design

The digital solution was conducted using a collaborative, pragmatic approach, influenced by Greenhalgh et al’s [17] Diffusion of Innovations Model, to develop a solution that could be implemented at a national scale. We used a series of stakeholder meetings scheduled as part of the Veterans’ MATES program to map out context and understand adopters’ (ie, GPs’) practices and preferences. The meetings involved funder (DVA) representatives, clinicians, veteran representatives, and information technology professionals.

To develop the solution, some particularities of the Australian health system and context were considered relevant, in particular:

- Reliance on primary care providers: The GP is the gatekeeper of the Australian health care system. About 84% of Australians see a GP every year, and 77% of patients have a preferred GP [18].
- Geographical distribution: GPs responsible for Australian veterans are located in all parts of the country. There are only a few GPs specialized in veteran care; most professionals have less than a handful of veterans under their care.
- Technological readiness in primary care: Use of electronic health records in primary care has been widespread in Australia for at least 10 years [19]. Additionally, secure messaging infrastructure is well established for receiving laboratory test results.

The proposed solution is an adaptation of the 3 steps used by Veterans’ MATES interventions, suited for a digital medium. To identify individuals at risk of medication-related harm, the solution uses a set of algorithms to extract information from claims data (services and medicines) indicating phenotype (which conditions affect the patient based on the resources they use). These algorithms identify patients at risk, either due to long-term conditions, medicine use, or current events such as medicine discontinuation.

To create the electronic messages, patient information extracted from the claims database is embedded in a template to create an audit and feedback document designed to promote recognition of patient risk. The document uses behavior change techniques, including prompts, goal setting, discrepancy between current behavior and goal, information about health consequences, and feedback on behavior; all of these techniques have been shown to improve intervention usefulness [16].

Documents are created as PDF documents, encrypted, and embedded in a Health Level Seven (HL7) version 2 file using internally developed software. Audit and feedback documents may contain complex graphical elements and may change significantly according to patients’ conditions and suggested recommendations. Therefore, we chose to initially develop documents as HTML pages, which are then converted to PDF format.

Finally, our investigation suggested the suitability of using an existing secure message infrastructure to reach GPs. Encrypted HL7 messages are sent to GP offices using a third-party provider and then decrypted by the clinical software and incorporated into the GP workflow. This solution adheres to many determinants of innovation diffusion identified in [17], as shown in Table 1.

**Table 1:** Determinants of innovation diffusion and predicted advantages of proposed solution.

Determinant of innovation	Predicted advantages of proposed solution
Relative advantage	Electronic messages are easier to read and act upon and less cumbersome than other communication means, such as printed materials or telephone communication
Compatibility	The solution uses communication infrastructure already being used to receive laboratory test results, with minimal additional impact on clinician workflow
Complexity	The solution can be described by the three main processes (patient identification, message tailoring, and secure delivery), which are understood by all stakeholders
Trialability	The solution was trialed in 3 small pilots and 1 randomized controlled trial before large-scale adoption
Risk	The solution has a relatively low cost and builds upon a 15-year program, reducing risk
Task issues	The solution is embedded in current workflow, with minimal task disruption
Augmentation/support	Each message is data driven, meaning it offers information related to a unique patient, also providing clear and unambiguous recommendations

### Feasibility Studies

The most important implementation risk identified during the initial stakeholder meetings was that the intervention could be perceived as intrusive and disruptive to GP workflow. To mitigate this risk, the solution was trialed in 3 sequential small-scale pilots, taking place in April, July, and September 2019. The main goals of the pilots were the following:

1. Evaluate the technical feasibility. We measured the proportion of messages acknowledged as successfully received.
2. Reduce the risk of disrupting GP work practices. GPs involved in the pilot could get in touch via a support email, website, and telephone. Additionally, we sent an invitation to an online survey containing 16 questions about usability and satisfaction.

The first pilot was planned as an opt-in trial, and GPs were invited to participate by email (Multimedia Appendix 1). The second pilot was planned as an opt-out trial, and GPs were sent an email explaining the study and offering the opportunity to be removed from the list. The final trial was planned as usual service and preceded by a mailed information leaflet (Multimedia Appendix 2).

### Trial Design

To test the influence of mode of delivery on the effectiveness of audit and feedback interventions, we performed a parallel, cluster randomized trial of a computer-delivered intervention to reduce inappropriate gabapentinoid prescription. The trial was designed to compare the post-delivered intervention (usual intervention as concurrent control) with the computer-delivered intervention. Since the intervention targets GPs who may have multiple veteran patients, we adopted a cluster design whereby a GP received information for all of their patients in the intervention by the same mode.

The intervention delivered via postal mode has been shown effective in translating evidence in different domains [20] including promoting medicine review [21], osteoporosis screening [22], uptake of health services [23], reducing inappropriate proton pump inhibitor use [24], and hypnotic use for insomnia [25].

### Participants

To be eligible for participation, both veterans and their primary GP had to be eligible for the digital intervention. Eligible veterans comprised active DVA clients that had 2 or more gabapentinoid (either pregabalin or gabapentin) prescriptions in a 4-month period (October 2019 to January 2020). Veterans were also required to be resident in Australia, living in the community setting (ie, not residing in aged care or other long-term care facilities), and to not have previously requested exclusion from Veterans’ MATES interventions for any reason.

GPs were eligible if they were identified as the primary GP of one or more Australian veterans, and at least one of the veterans was eligible for the intervention. Participant GPs were excluded if they did not have installed capacity to receive secure electronic messages from our partner message provider (HealthLink Group Limited) or if they had previously requested exclusion from Veterans’ MATES interventions for any reason.

To determine the primary GP for a given veteran, we developed an algorithm based on prescriptions and outpatient services provided. Providers were scored based on the number of prescriptions and services provided, and weighted based on recency of services to account for veterans changing providers.

### Setting

The trial was conducted across all Australian states and territories. We determined patient eligibility by querying the DVA claims database. Outcome data including service provision and medicine dispensing were collected from the DVA claims database.

### The Intervention

GPs in the intervention arm received the intervention exclusively in a digitally delivered format. It was sent via secure message infrastructure directly to the GP’s clinic. Once received by the practice, it is reviewed by a practice manager of the GP and assigned to the appropriate patient. Once it is assigned, it can be accessed in the electronic health record alongside pathology reports and referral letters.

GPs in the usual care arm received the intervention by postal service. This delivery contains both the audit and feedback documents (for all selected veteran patients) and the educational materials (including a copy of the material targeted at veterans).

**Figure 1:**
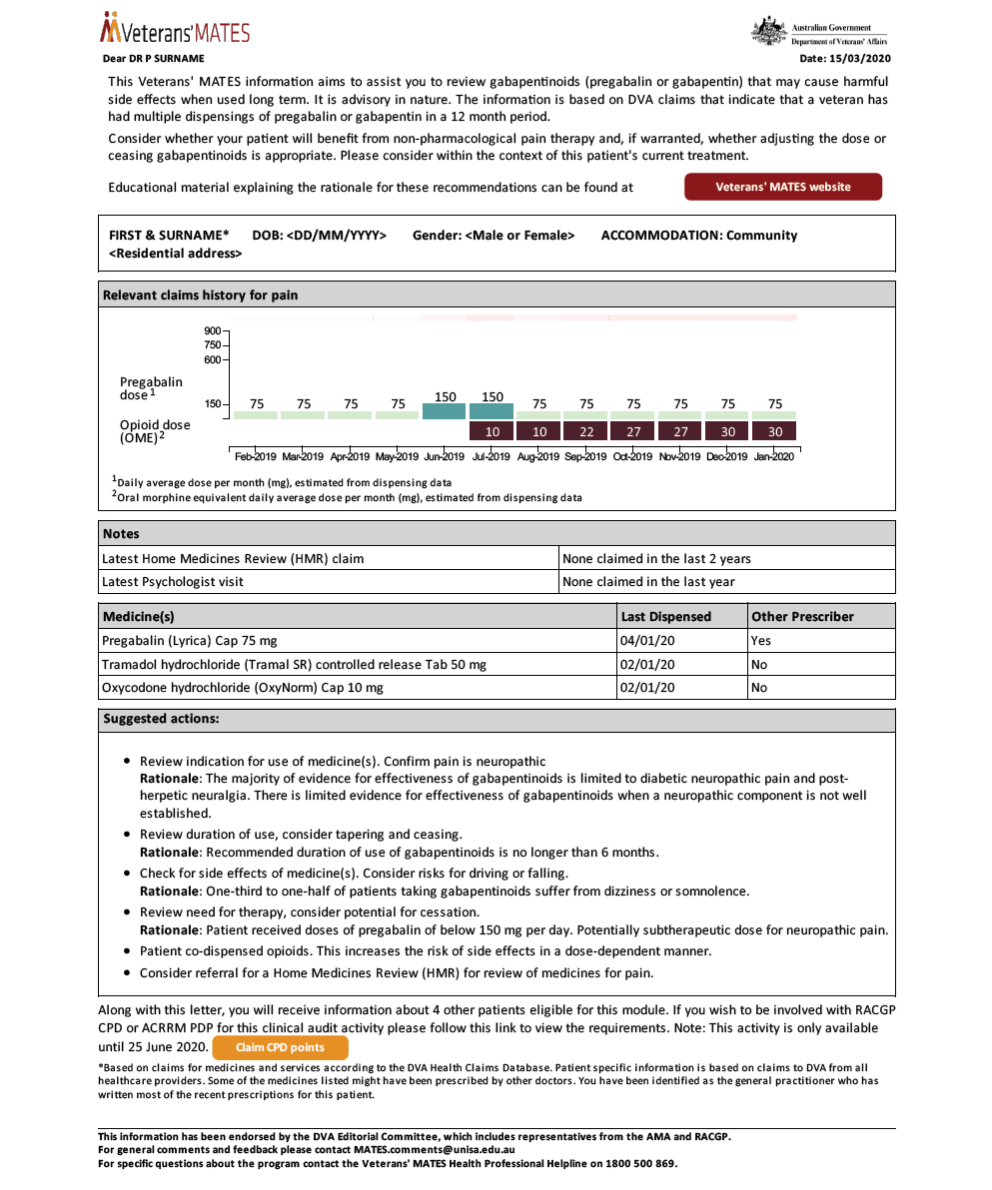
Example of the intervention delivered to general practitioners (digital version).

Both sets of materials contained the same theoretical content and personal information. Due to feedback from users, the digitally delivered intervention was slightly modified to user workflow. Since we could not deliver general educational documents to the health record, the audit and feedback document was enhanced to contain a link to the online educational material (see Figure 1). Additionally, the single letter containing multiple patients was segmented into one electronic document per patient. Finally, a color chart was added at the top of the electronic document to highlight different prescription patterns and help GPs prioritize patients when receiving multiple documents.

Veterans in both the intervention and usual care arms received educational material by post. The material can be found on the Veterans’ MATES web page [26].

### Enrolment and Randomization

Following the eligibility criteria, all GPs acting as the main care provider for an eligible patient (current gabapentinoid use) were considered for recruitment. We excluded all practitioners not found in the partner’s (Healthlink Group Limited) provider directory, as they would be unable to receive secure electronic messages. All eligible GP and patient pairs were included in the study sample.

GPs were randomized 1:1 to intervention or usual care. Randomization was block stratified by number of veterans under care. Randomization numbers for each GP were computer generated by a statistician who was not involved in enrolment. Due to the highly automated nature of the intervention and data collection (claims data), no further masking procedures were performed.

An ethics protocol for the study was approved by the University of South Australia Human Research Ethics Committee (ethics protocol P203/04) and the Australian Government Department of Defence and Veterans’ Affairs Human Research Ethics Committee (E016/007).

### Outcomes

The primary outcomes were the change in average gabapentinoid prescription during the study period, standardized as multiples of the defined daily dose (DDD) per day, and the proportion of veterans visiting a psychologist for the first time. Primary outcomes were evaluated at 6 and 12 months.

Since the dosing of pregabalin and gabapentin are different, we calculated DDD for each medicine and summed the results. To remove the influence of extreme stockpiling and dispensing data errors, patients with a DDD over 10 (10 times the defined daily dose) were removed from analysis. The DDD was created by the World Health Organization (WHO) as a comparative unit of medicine use [27]. In this study, it allows the comparison of different gabapentinoids, such as gabapentin and pregabalin. The average daily DDD was calculated as per the following formula:



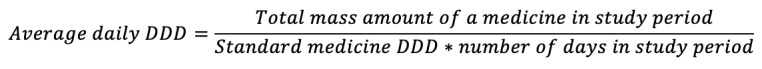



The total mass amount was determined according to all claimed prescriptions of gabapentin and pregabalin in the 3 months prior to the intervention (January 3, 2020, to April 2, 2020) and in the 6 months (July 3, 2020, to October 2, 2020) and 12 months (January 3, 2021, to April 2, 2021) following the intervention.

Secondary outcome was the time to the first visit (face-to-face, telephone, or video) with the primary provider. All outcome measures pertain to the individual (patient).

We conducted a secondary analysis that can be divided into two parts. First, we evaluated the overall intervention impact by measuring changes in average gabapentinoid DDD before and after the intervention. Furthermore, we evaluated whether the dose of gabapentinoid (high, medium, or low) or concurrent use of opioids influenced the efficacy of the different modes of delivery. Veterans were considered to be on a high dose if the average DDD in at least one month of the selection period was >2. Veterans were considered to be on a low dose if the average DDD in every month of the selection period was <0.25. Values between those two values were considered to be a medium dose.

### Statistical Methods

We analyzed data from services and medicines claims for all enrolled patients who were alive at 12 months postintervention. To account for the cluster design, we analyzed the primary outcome using a linear mixed-effects model [28], with the GP as the grouping variable. The effect of mode of delivery on patients’ likelihood of visiting a psychologist was tested by logistic regression, also using GP as the grouping variable. The time to first GP visit was analyzed by survival analysis. Patients were considered to have the “event” if they had an appointment with the targeted GP. Events were right censored at 3 months (92 days). The relative effect of digital mode versus postal mode was evaluated by a Cox proportional hazards model, with the GP as cluster variable. Secondary analysis was performed by univariate linear mixed-effects model, with the GP as grouping variable. For all hypothesis tests, we considered a 95% CI (*P*≤.05). All analysis was performed in Python 3.7 (The Python Software Foundation). The main statistical libraries used were Statsmodels (version 0.12) [29] and Lifelines (version 0.25.11) [30].

### Availability of Data and Material

The data that support the findings of this study are available from the Australian Government DVA but restrictions apply to the availability of these data, which were used under license for this study, and so are not publicly available.

## Results

### Feasibility Studies

For the first pilot, a convenience sample of 75 GPs were sent an email invitation to participate (opt-in), and 5 GPs agreed to be included. For the second pilot, we selected a convenience sample of 20 GPs who could opt out of the pilot. For the third pilot, 189 messages were sent to GPs who had not participated previously. We received 6 survey responses, and all responders evaluated the usability as good (easy to read, correct information) and were either likely or very likely to continue to subscribe to future interventions. We received a single letter advising a patient had recently switched medical providers. Given the lack of negative feedback and positive survey responses, the project leadership considered the pilot successful and the intervention feasible, and approved the randomized controlled trial.

### Cluster Randomized Controlled Trial

**Figure 2:**
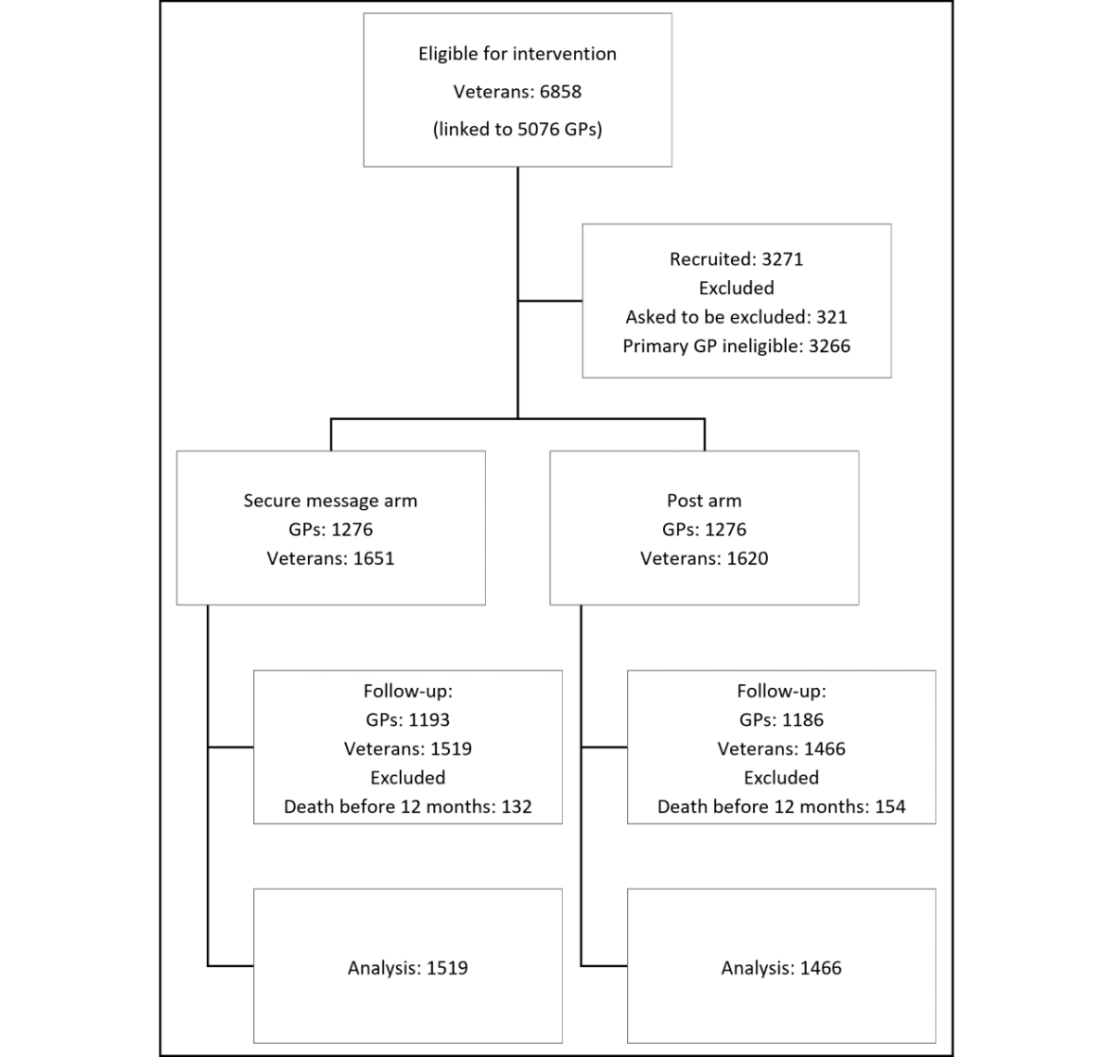
CONSORT (Consolidated Standards of Reporting Trials) flowchart. GP: general practitioner.

A total of 3271 veterans were considered eligible for the intervention, and 2552 GPs were identified as their main care providers (Figure 2). After randomization, the intervention was successfully delivered in March/April 2020. The postal intervention was sent to GPs on March 19, 2020. The computer-delivered intervention was delivered in three waves (on March 23, March 25, and April 2, 2020).

Veterans randomized to either intervention arm had a similar demographic profile (Table 2). The patterns of gabapentinoid and opioid use were also similar.

**Figure 3:**
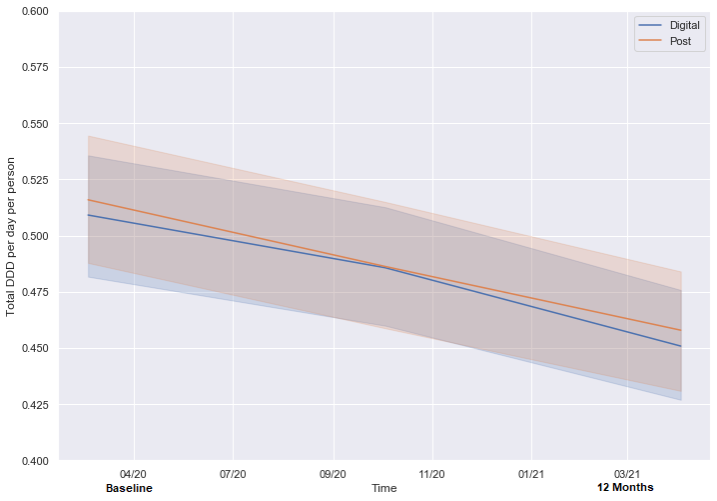
Average daily DDD by intervention group. DDD: defined daily dose.

By the end of the study, both intervention groups had a significant reduction in gabapentinoid dispensing, as measured by the change in average daily DDD from baseline to 12 months (digital: mean reduction of 0.058, SD 0.38, or 11.2%, *P*=.004; postal: mean reduction of 0.058, SD 0.37, or 11.2%, *P*=.001). Figure 3 shows the trends in DDD before and after the intervention.

**Table 2:** Clinical and demographic data at baseline.

Baseline data			Postal intervention	Digital intervention
Number of participants			1466	1519
Age (years), mean (SD)			76.1 (14.6)	76.1 (14.5)
Male, n (%)			853 (58)	883 (58)
**Gabapentinoid dose at baseline, n (%)**				
	High	41 (3)		34 (2)
	Medium	1188 (81)		1213 (80)
	Low	237 (16)		272 (18)
Concurrent opioid use, n (%)			590 (40)	636 (42)

We found no difference between digital and postal mode of delivery in reduction of gabapentinoid volume at 6 or 12 months (Table 3). A greater proportion of veterans in the digital intervention group saw a psychologist in the following 12 months (*P*=.02). Digital intervention promoted a small but statistically significant (*P*=.04) effect of earlier GP visits postintervention.

Veterans were segmented according to dose and concurrent opioid use. Consistent with the results of the primary analysis, no differences were found between the digital and postal interventions in any subgroup analysis. Dose reduction was more pronounced in high-dose gabapentinoid users, and there was no observed reduction in the average dose of low-dose users in either arm.

**Table 3:** Primary and secondary outcomes, by intervention arm.

Outcomes	Postal	Digital	*P* value
Average defined daily dose change (baseline to 6 months)	–0.030	–0.023	.61
Average defined daily dose change (baseline to 12 months)	–0.058	-0.058	.98
Percentage of new psychologist visits (baseline to 6 months)	1.0	1.3	.75
Percentage of new psychologist visits (baseline to 12 months)	2.0	3.8	.02^a^
Hazard ratio for general practitioner visit within 90 days (95% CI)	0.92 (0.85-0.99)	1 (reference)	.04^a^

^a^*P*<.05.

## Discussion

### Principal Findings

In this study, we present the successful migration of a paper-based national behavior change intervention into a digital intervention. After a careful scaling of the intervention, ample communication, and stakeholder support, we were able to perform a large-scale randomized controlled trial covering all Australian states. The trial showed that both paper and digital versions of an intervention composed of education and audit and feedback was effective in reducing gabapentinoid prescriptions for an Australian population. Additionally, it showed that the digital intervention is equivalent to paper in changing prescription patterns.

This study is one of the first to test the effect of mode of delivery in a large-scale, precision public health intervention. The use of a digital medium of delivery has several advantages over conventional interventions, including the capacity for improved personalization and precision; improved automation; use of predictive analytics for targeting; data analytics; and improved interaction [31]. However, any new intervention may have unforeseen consequences, and requires testing as with any other new technology [32]. The digital media and paper version had similar effectiveness for affecting medicine use, but this study provides emerging evidence that digital intervention may be superior for services that require referral.

Digital delivery changes how participants interact with and experience the intervention. Integration with the patient electronic health record reduces the effort required to create new patient requests, such as actively inviting patients to a follow-up appointment. Therefore, the incorporation of the intervention in a clinician’s workflow may explain the increased number of GP visits and psychologist referrals after the digital intervention when compared to the usual intervention. Creating a request to follow up a patient is easy to implement and unlikely to cause significant disruption. In contrast, reducing the dose of gabapentinoids, commonly indicated for pain, requires careful consideration and close patient contact and participation. Our results suggest that both postal and digital interventions are effective in promoting dose change, but it is possible that the digital medium advantage lies in creating triggers that can be easily followed.

The timing of this study is an important limitation of this study, as intervention delivery coincided with the initial restrictions implemented in Australia in response to the COVID-19 pandemic in March 2020. The week of the intervention, several policies to restrict gatherings and reduce risk of contagion were enacted [33], which influenced some of the metrics used in this study. Medicine dispensing was likely affected, with stockpiling occurring and a temporary lack of access. Additionally, many clinics were closed to avoid waiting room risks, and appointments via telehealth were funded by the Department of Health. This may have influenced intervention effectiveness, as the opportunity to adjust therapy was reduced; however, it is unlikely to have affected the assessment of mode of delivery as both arms of the trial would have been equally affected by the COVID-19 restrictions. Postal mail services were fully maintained during restrictions.

This study also provides a foundation for further research aimed at improving the effectiveness of audit and feedback in public health digital interventions. The effect size of conventional and digital audit and feedback interventions is usually small [1,7], and a clear methodology to improve effect remains an open question. Effect may be influenced by factors related to the recipient (eg, GP), behavior, or content and delivery of the intervention [34]. Using digital media enables nationwide programs such as Veterans’ MATES to contribute to such research by creating repeated interventions at lower cost, with greater speed and precision.

### Conclusion

This study showed a digitally delivered professional behavior change intervention had comparable effectiveness to a postal intervention and superior efficacy for referral services. Given the logistical benefits of digital delivery in nationwide programs (cost, speed, and precision), the results encourage exploration of this mode in future interventions.

## Appendix

Multimedia Appendix 1 Opt-out letter.

Multimedia Appendix 2 General practitioner communication campaign to inform about digital intervention.

Multimedia Appendix 3 CONSORT-eHEALTH checklist (V 1.6.1).

## Abbreviations

DDD: defined daily dose
DVA: Department of Veterans’ Affairs
GP: general practitioner
HL7: Health Level Seven
MATES: Medicines Advice and Therapeutics Education Services
RCT: randomized controlled trial
WHO: World Health Organization
