# Expression of Concern: Implementation and Evaluation of a Digitally Enabled Precision Public Health Intervention to Reduce Inappropriate Gabapentinoid Prescription: Cluster Randomized Controlled Trial

**DOI:** 10.2196/80518

**Published:** 2025-07-17

Following publication of this article [1] concerns have been raised regarding the process by which participant data was collected for use in the study and the extent of informed consent granted by participants during the data collection process by the Australian Department of Veterans’ Affairs.

The JMIR Editorial Office will investigate these issues in accordance with journal policies on ethical approval and by adhering to Committee on Publication Ethics (COPE) guidelines in seeking to resolve the matter.

In the interim, the JMIR Editorial Office issues this Expression of Concern. Further information will be provided in due course.
