# Supplementary figures and images for: Implementation and Evaluation of a Digitally Enabled Precision Public Health Intervention to Reduce Inappropriate Gabapentinoid Prescription: Cluster Randomized Controlled Trial
Source: J Med Internet Res. 2022 Jan 10;24(1):e33873. doi: 10.2196/33873 (PMC8787661; doi:10.2196/33873)

# Appendix 2 – GP communication campaign to inform about digital intervention


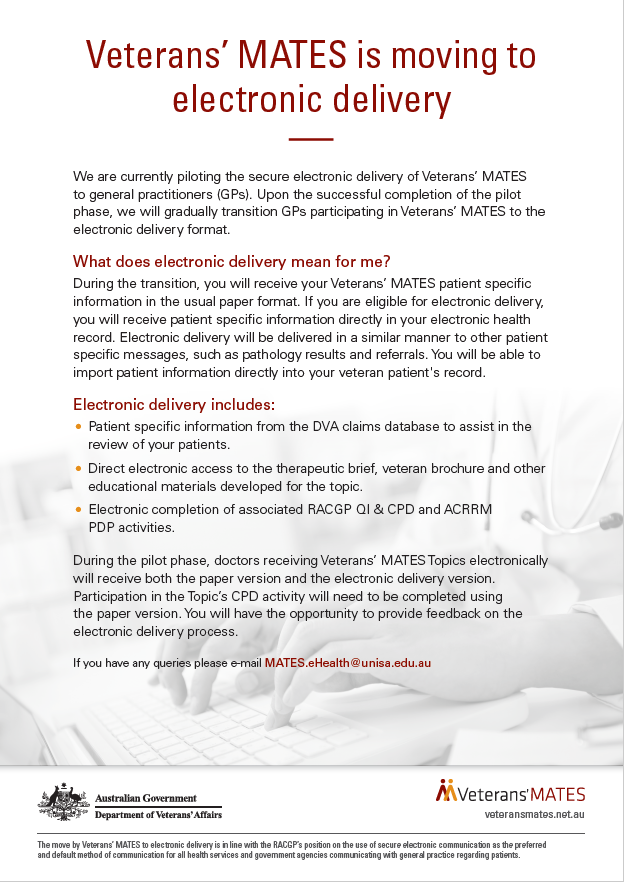

Supplement: Multimedia Appendix 2 [file jmir_v24i1e33873_app2.docx]
