# Supplementary material for: Implementation and Evaluation of a Digitally Enabled Precision Public Health Intervention to Reduce Inappropriate Gabapentinoid Prescription: Cluster Randomized Controlled Trial
Source: J Med Internet Res. 2022 Jan 10;24(1):e33873. doi: 10.2196/33873 (PMC8787661; doi:10.2196/33873)
Supplement: Multimedia Appendix 1 [file jmir_v24i1e33873_app1.docx]

# Appendix 1 - OPT OUT LETTER

Dear Dr FIRSTNAME SURNAME

The Department of Veterans’ Affairs (DVA) has been delivering the *Veterans’ MATES*^1^ program since 2004, with the aim of improving the use of medicines and related health services in the veteran community.  As part of the arrangements for provision of the program, DVA has requested UniSA to develop the electronic delivery of Veterans’ MATES education material.  In line with the RACGP’s position on the use of secure electronic communication as the preferred method of communication,^2^ Veterans’ MATES is gradually moving to electronic delivery in 2019. A preliminary pilot of electronic delivery of *Veterans’ MATES* was carried out in April 2019.

As you have received the latest *Veterans’ MATES* topic on insomnia, released 21 June 2019, we would like to invite you to be involved in the second phase of piloting the secure electronic delivery of *Veterans’ MATES* directly to General Practice software scheduled for July.

**What will the pilot involve?**

You would have received your patient specific information and educational material for the Insomnia topic in the usual manner. This is to ensure you can participate in the CPD activity as per normal.  By mid-July, you will also receive the topic information electronically in the same way you receive other patient and clinical messages, such as pathology results and referrals. You can choose to review your patients as per normal or by using the information you received electronically. You will be also able to import the information you received electronically directly into your veteran patient's record.

Doctors involved in the pilot will be invited to provide feedback via a short survey distributed towards the end of July.

If you prefer not to be involved in the pilot, please reply ‘NO TO PILOT’ in the subject line.

If you would like further information, please do not hesitate to e-mail us on [MATES.eHealth@unisa.edu.au](mailto:MATES.eHealth@unisa.edu.au) with details of your query.

Yours sincerely

Professor Libby Roughead

Executive Director

Veterans' MATES Program

Quality Use of Medicines and Pharmacy Research Centre

University of South Australia

^1^Medicines Advice and Therapeutics Education Services

^2^The move by *Veterans’ MATES* to electronic delivery is in line with the RACGP’s position on the use of secure electronic communication as the preferred and default method of communication for all health services and government agencies communicating with general practice regarding patients.
